# Neural stem cell phenotype of tanycyte-like ependymal cells in the circumventricular organs and central canal of adult mouse brain

**DOI:** 10.1038/s41598-020-59629-5

**Published:** 2020-02-18

**Authors:** Eriko Furube, Haruna Ishii, Yuri Nambu, Erkin Kurganov, Sumiharu Nagaoka, Mitsuhiro Morita, Seiji Miyata

**Affiliations:** 1 Department of Applied Biology, Kyoto Institute of Technology, Matsugasaki, Sakyo-ku, Kyoto 606-8585 Japan; 2 Department of Anatomy, Faculty of Medicine, Toho University, Omori, Ota-ku, Tokyo 143-8540 Japan; 3 Department of Biology, Graduate School of Science, Kobe University, Kobe, Japan

**Keywords:** Adult neurogenesis, Neural stem cells

## Abstract

Tanycyte is a subtype of ependymal cells which extend long radial processes to brain parenchyma. The present study showed that tanycyte-like ependymal cells in the organum vasculosum of the lamina terminalis, subfornical organ and central canal (CC) expressed neural stem cell (NSC) marker nestin, glial fibrillar acidic protein and sex determining region Y. Proliferation of these tanycyte-like ependymal cells was promoted by continuous intracerebroventricular infusion of fibroblast growth factor-2 and epidermal growth factor. Tanycytes-like ependymal cells in the CC are able to form self-renewing neurospheres and give rise mostly to new astrocytes and oligodendrocytes. Collagenase-induced small medullary hemorrhage increased proliferation of tanycyte-like ependymal cells in the CC. These results demonstrate that these tanycyte-like ependymal cells of the adult mouse brain are NSCs and suggest that they serve as a source for providing new neuronal lineage cells upon brain damage in the medulla oblongata.

## Introduction

During the last two decades, it has been widely established that neural stem cells (NSCs) exist in the subventricular zone (SVZ) and the subgranular zone (SGZ), where new neurons and glial cells are constantly produced in adult brain^1–3^. The SVZ locating along the ependymal layer generates new interneurons in the olfactory bulb, which are important for short-term olfactory memory and flexible olfactory associative learning^4–7^. The SGZ in the hippocampus gives rise to new dentate granule cells, which implicate in long-term spatial memory and pattern separation cognitive functions^5,8,9^. It is also reported that stroke stimulates progenitor proliferation and leads to long-term changes in the NSC niche in the SVZ of adult rodents^10^. In addition, NSCs may contribute to repair not only neuronal cell loss but also glial dysfunction, for example chronic demyelinating disease^11^. These studies indicate that NSCs in the adult brain are specialized for region-specific functions and moreover have repair functions against brain damages.

Although research on adult mammalian NSCs has focused almost exclusively on two areas, the SGZ and SVZ, several recent studies reported the existence of NSCs in tanycytes of the hypothalamus^12,13^. Tanycyte is a subtype of ependymal cells that resembles radial glia-like cells, lines the ventricular wall and extends long radial process from their cell body^14^. Tanycytes have mainly been described in the median eminence (ME) and the arcuate nucleus (Arc) of the hypothalamus and are classified into four types, α 1, α 2, β 1, and β 2, depending on the position, morphology and microstructure^14,15^. In young adult mice, β-tanycytes in the ME are shown as NSCs that generate new neurons and glial cells^13^. The suppression of neurogenesis in the ME of young adult mice results in increase in body weight by reducing metabolic activity^13^. In fully adult mice, however, α-tanycytes in the Arc, but not the ME, have the potential to self-renew, produce astrocytes and neurons and proliferate in response to the fibroblast growth factor-2 (FGF-2) and epidermal growth factor (EGF)^12^. Thus, the mediobasal hypothalamic regions apparently have different functions from the SVZ and SGZ, in other words, they possess the region-specific function that de novo neurogenesis is responsible for the plastic control of energy balance such as food intake and body adiposity^13^.

The circumventricular organs (CVOs) have permeable fenestrated capillaries unlike those in the rest of the brain, since they lack a typical blood-brain barrier or tight junction proteins^16^. They consist of the organum vasculosum of the lamina terminalis (OVLT), subfornical organ (SFO), ME and area postrema (AP)^17,18^. The CVOs are the blood-brain interface to exchange the information between brain parenchyma cells and blood circulation^17–19^. Recently, it was shown that NSCs existed in the OVLT, SFO and AP of adult mouse brain^20,21^. In our previous study, moreover, we characterized expression of marker proteins and vascular endothelial growth factor-dependent increase and inflammation-dependent decrease in proliferation in astrocyte-like NSCs of the CVOs^22^. Moreover, NSCs are shown to possess the potential to produce oligodendrocytes and a small number of neurons and astrocytes in the neighboring brain regions as well as the CVOs^22^. NSCs line the lumen of the neural tube during development and the corresponding structure in the adult, the ventricular system is suggested to constitute a NSC niche. However, evidences whether or not tanycyte-like ependymal cells in the CVOs and central canal (CC) of the medulla oblongata are NSCs remain wholly insufficient. Moreover, it is supposed that putative NSCs, tanycyte-like ependymal cells, in the CC function as an endogenous repair system for broad medullary regions, since the CC runs throughout the entire length of the medulla oblongata and spinal cord and connected with the forth ventricle^23^.

To directly address these questions, we aimed to elucidate whether or not tanycyte-like ependymal cells in the CVOs and CC of the medulla oblongata are NSCs or tanycytes and tanycyte-like ependymal cells in the adult brain have NSC niche. Ependymal cells in the CVOs are reported to have tanycyte features, since they extend long and slender fibers from the ependymal cell bodies to form a dense network surrounding fenestrated capillaries^24–26^. In the present study, we showed that tanycyte-like ependymal cells in the OVLT, SFO and CC are NSCs by the immunohistochemistry of NSC marker proteins, *Nestin-CreERT2/CAG-CAT*^loxP/loxP^*-EGFP* transgenic mouse and growth factor-dependent proliferation. Moreover, collagenase-induced small medullary hemorrhage facilitated proliferation of tanycyte-like ependymal cells in the CC of the medulla oblongata. Neurosphere assay showed that tanycyte-like ependymal cells in the CC of the medulla oblongata had capability for self-renewing and could differentiation into astrocytes and oligodendrocytes. These result demonstrates that tanycyte-like ependymal cells existing in the CVOs and CC of the medulla oblongata are NSCs and suggests that NSCs in the CC are able to supply new neuronal lineage cells to broad regions of the medulla oblongata after injury.

## Results

### Tamoxifen-induced enhanced green fluorescent protein (EGFP) expression in tanycyte-like ependymal cells in adult brains

**Figure 1:**
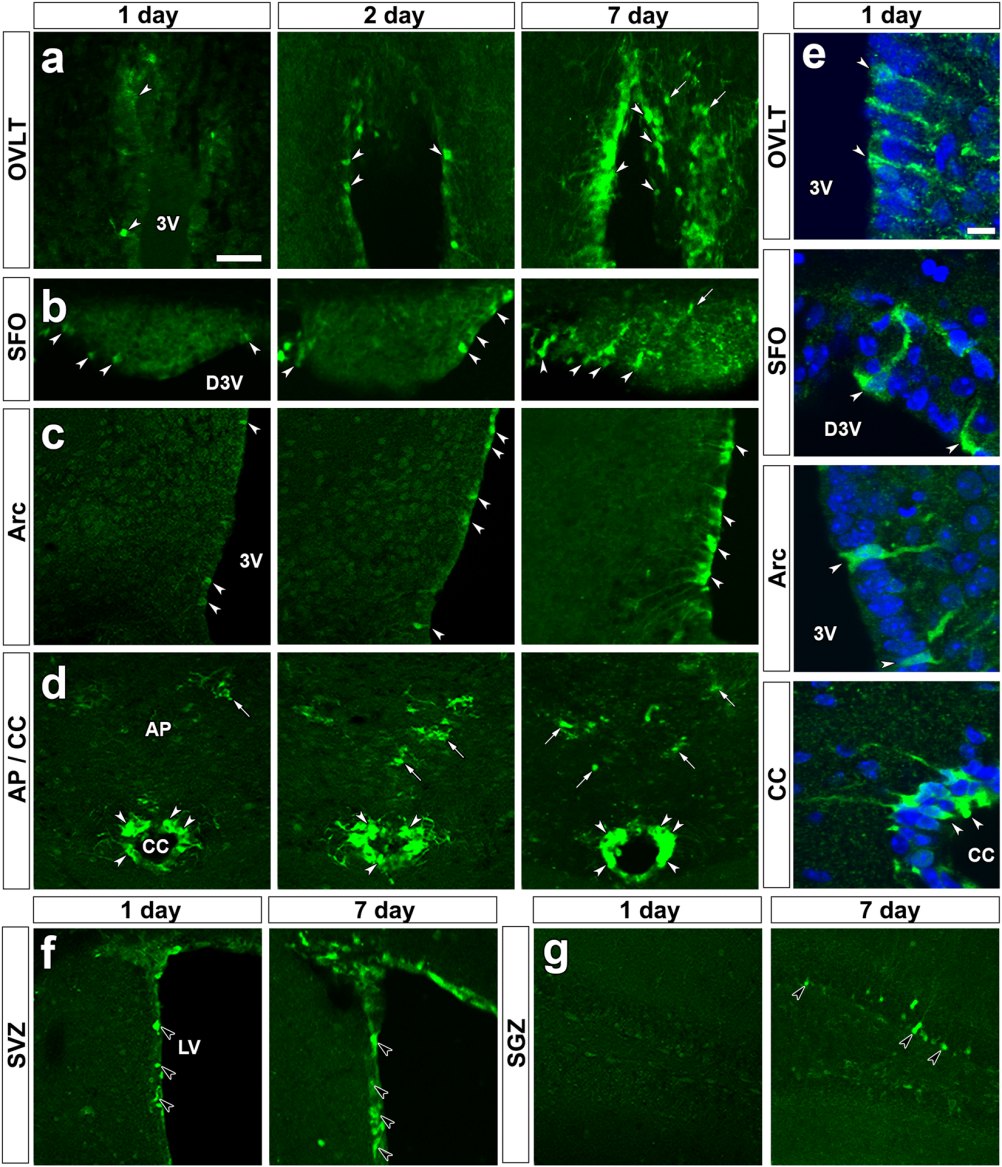
Time course changes in EGFP^+^ cells in the brain of adult *Nestin-CreERT2/CAG-CAT*^loxP/loxP^*-EGFP* mice. The transgenic mice fixed at 1, 2 and 7 days after a single intraperitoneal administration of 180 mg/kg tamoxifen. Low magnification views showed the presence of EGFP^+^ tanycyte-like ependymal cells (open arrowheads) in the OVLT (**a**), SFO (**b**) and CC (**d**) and EGFP^+^ tanycytes (open arrowheads) in the Arc (**c**) at 1, 2 and 7 days after the tamoxifen treatment. High magnification views with nuclear dye DAPI revealed that EGFP^+^ tanycytes/tanycyte-like ependymal cells extended long cellular processes to brain parenchyma from their cell bodies (**e**). EGFP expression in astrocyte-like NSCs (arrows) were weak or faint in the OVLT (**a**), SFO (**b**) and AP (**d**) at 1 and 2 days after the tamoxifen treatment, but it became stronger at 7 days. EGFP^+^ NSCs (solid arrowheads) were observed in the SVZ (**f**) at 1 and 7 days after the tamoxifen treatment, but those in SGZ (**g**) were detected only at 7 days after the tamoxifen treatment. Scale bars 50 μm. 3V, third ventricle; D3V, dorsal third ventricle; LV, lateral ventricle.

**Figure 2:**
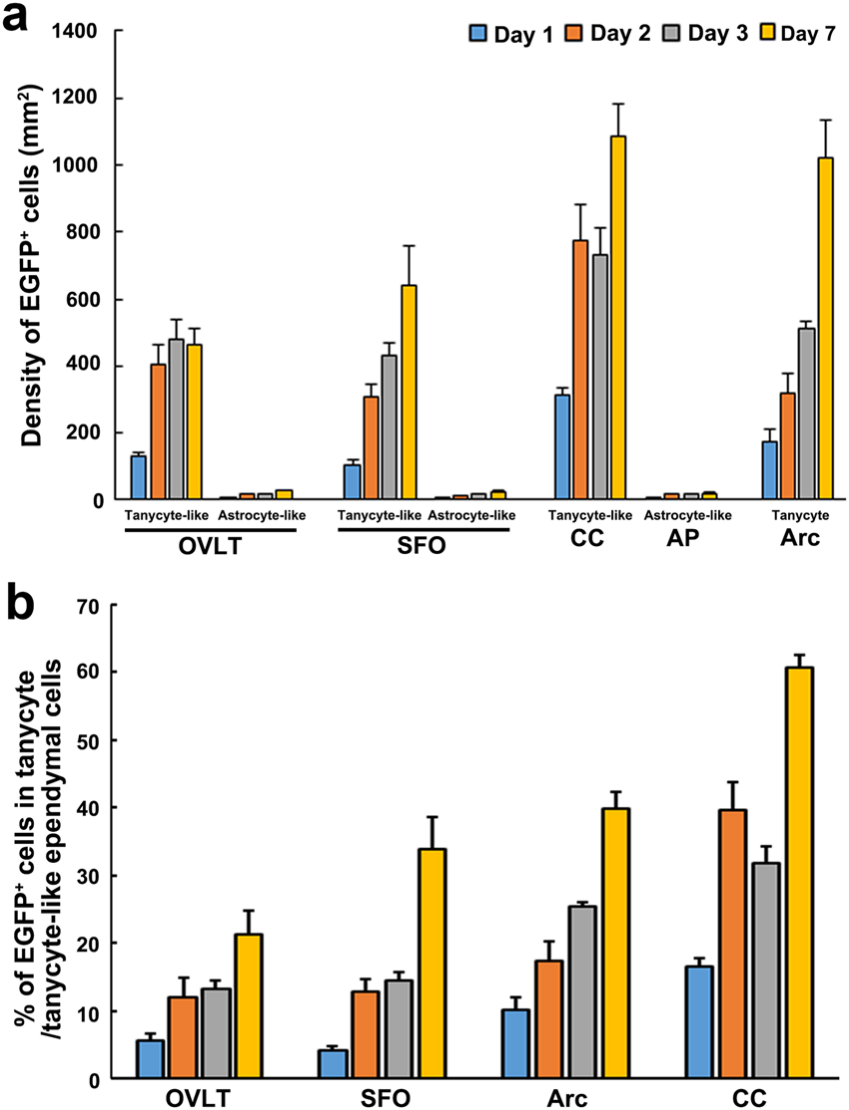
The quantification of the density and percentage of EGFP^+^ tanycyte/tanycyte-like ependymal cells in the brain of adult transgenic mice. The transgenic mice fixed at 1, 2, 3 and 7 days after a single intraperitoneal administration of 180 mg/kg tamoxifen and EGFP^+^ cells were quantified. The density of EGFP^+^ tanycyte-like ependymal cells increased as elapsed days after the tamoxifen treatment (**a**). The percentages of EGFP^+^ cells in tanycyte/tanycyte-like ependymal cells increased as elapsed days after the tamoxifen treatment (**b**). Data shown as mean ± s.e.m. from 4 animals.

It has been reported that tanycytes are present in the ME and Arc^15,27^ and tanycyte-like ependymal cells exist in the OVLT, SFO^24–26^ and CC of the medulla oblongata^22^ of adult mouse brain. Nestin is widely used as a specific marker for NSCs^28,29^. A time course change in the appearance of EGFP^+^ cells in *Nestin-CreERT2/CAG-CAT*^*loxP/loxP*^*-EGFP* transgenic mice is shown in Fig. 1. The expression of EGFP was induced in tanycyte-like ependymal cells of the OVLT (Fig. 1a), SFO (Fig. 1b) and CC (Fig. 1d) and tanycytes of the Arc (Fig. 1c) at 1 day after a single administration of tamoxifen, while astrocyte-like NSCs did not show a prominent EGFP expression in the OVLT and SFO and AP. EGFP expression became stronger in tanycytes/tanycyte-like ependymal cells and astrocyte-like NSCs as the days passed. EGFP^+^ cells were observed in the Arc, but not the ME (Supplemenatry Fig. 1), which coincides with the previous report that NSCs are present only in the Arc of fully adult mouse^12^. High magnification confocal images showed that EGFP^+^ tanycytes/tanycyte-like ependymal cells extended long cellular processes from their cell body to brain parenchyma (Fig. 1e). The nuclei of tanycytes and tanycyte-like ependymal cells exclusively localized at ependymal layers that were densely labeled with nuclear dye 4,6-diamidino-2-phenylindole (DAPI). EGFP expression was specifically seen in the NSCs of the SVZ (Fig. 1f) and SGZ (Fig. 1g), well-accepted neurogenic regions, at 7 days after a single tamoxifen treatment, indicating reliability of the use of this transgenic mouse to detect adult NSCs. The quantitative evaluation of fluorescent images showed that the density of EGFP^+^ tanycytes and tanycyte-like ependymal cells in the CVOs, Arc and CC was high at 2 and 3 days after the tamoxifen treatment, but that of EGFP^+^ astrocyte-like cells was low (Fig. 2a). These data show unique property of the used transgenic mouse that tamoxifen-induced EGFP expression is faster or efficient in tanycytes and tanycyte-like ependymal cells. The EGFP^+^ percentage in tanycyte-like ependymal cells was 21.28 ± 3.41 in the OVLT, 33.88 ± 4.72 in the SFO and 60.63 ± 1.79 in the CC at 7 days after the tamoxifen treatment (Fig. 2b) and the EGFP^+^ percentage in tanycytes of the Arc was 39.75 ± 2.59. These results indicate that tanycyte-like ependymal cells in the CVOs and CC express a NSC marker nestin by using the *Nestin-CreERT2/CAG-CAT*^*loxP/loxP*^*-EGFP* mouse and show higher sensitivity to tamoxifen than astrocyte-like NSCs.

### Distribution of EGFP^+^ tanycyte-like ependymal cells in the CC of the medulla oblongata

**Figure 3:**
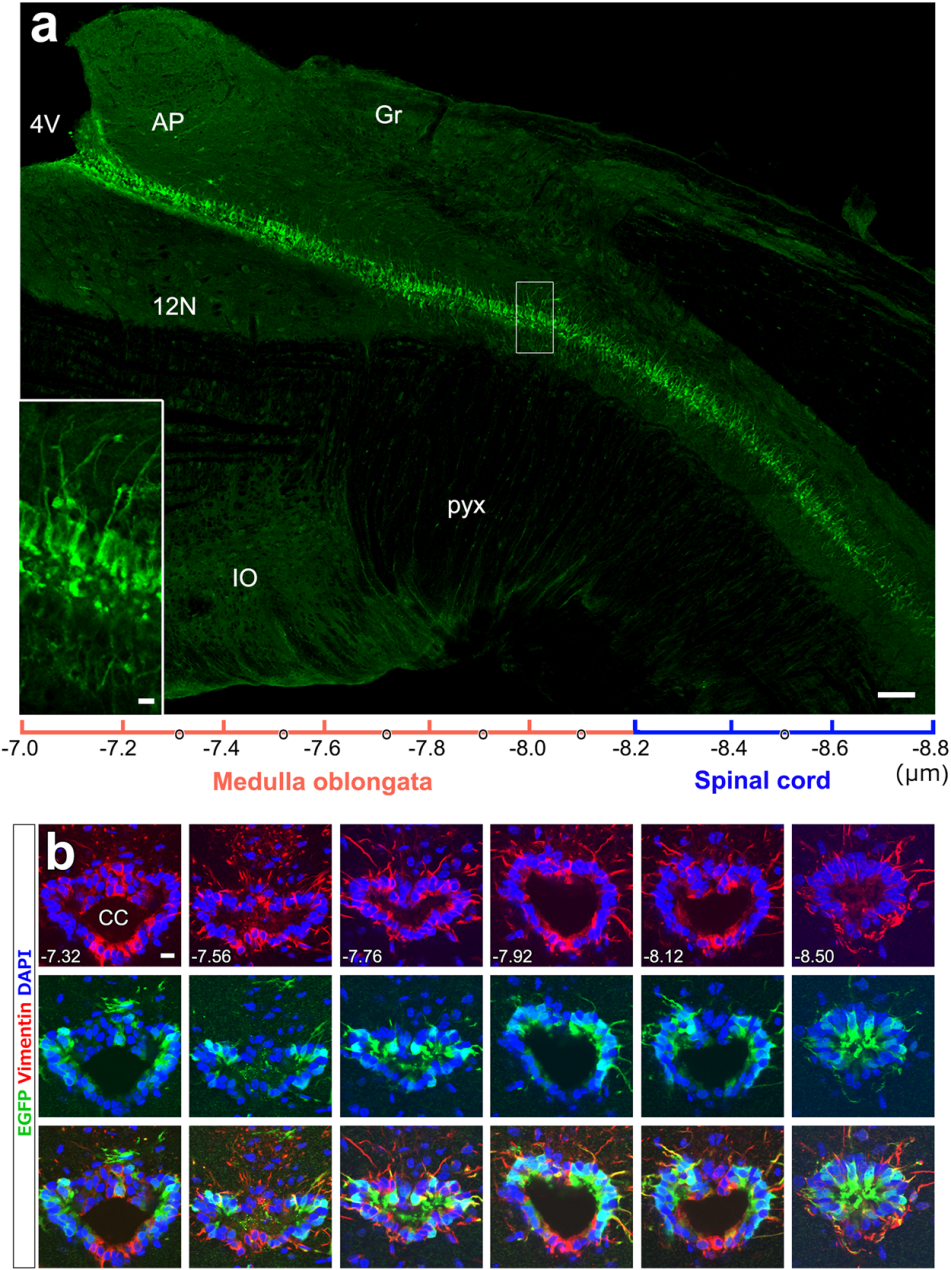
Distribution of EGFP^+^ tanycyte-like ependymal cells in the CC of the medulla oblongata of adult transgenic mouse. The transgenic mice fixed at 3 days after a single intraperitoneal administration of 180 mg/kg tamoxifen. A wide confocal image of sagittal sections showed that EGFP^+^ tanycyte-like ependymal cells existed continuously along the CC from the medulla oblongata to spinal cord (**a**). An enlarged image on the lower left indicated that EGFP^+^ tanycyte-like ependymal cells possess long cellular processes. Bottom scale indicated the distance from the bregma and the position examined for coronal images. Double labeling immunohistochemistry revealed that EGFP^+^ tanycyte-like ependymal cells in the wall of the CC extended long cellular processes (**b**). The number at right bottom in each panel indicates the position of bregama. Scale bars = 10 (an inset in a and b), 100 (**a**) μm. AP, area postrema; Gr, gracile nucleus; 12N, hypoglossal nucleus; pyx, pyramidal decussation; IO, inferior olive.

Although NSCs have been identified in tanycyte-like ependymal cells along the CC in the spinal cord^30,31^, it is not determined whether or not ependymal cells along the CC of the medulla oblongata exhibit tanycyte- and NSC-like properties. Therefore, EGFP expression and morphology of ependymal cells in the CC of the medulla oblongata and spinal cord of the transgenic mice were examined at 3 days after the tamoxifen treatment. The immunohistochemistry of sagittal sections revealed that EGFP^+^ ependymal cells were lining throughout the CC from the medulla oblongata to spinal cord (Fig. 3a). A high magnification view revealed that ependymal cells along the CC extended long cellular processes to medullary parenchyma. The immunohistochemistry of coronal sections using ependymal marker vimentin revealed the presence of EGFP^+^ ependymal cells (Fig. 3b). EGFP^+^ ependymal cells in the CC of the medulla oblongata and spinal cord were seen to extend long radial processes into brain parenchyma. These results indicate that nestin-expressing tanycyte-like ependymal cells distribute throughout the entire length of the medulla oblongata.

### Characterization of marker protein expression in tanycyte-like ependymal cells of the CVOs and CC

**Figure 4:**
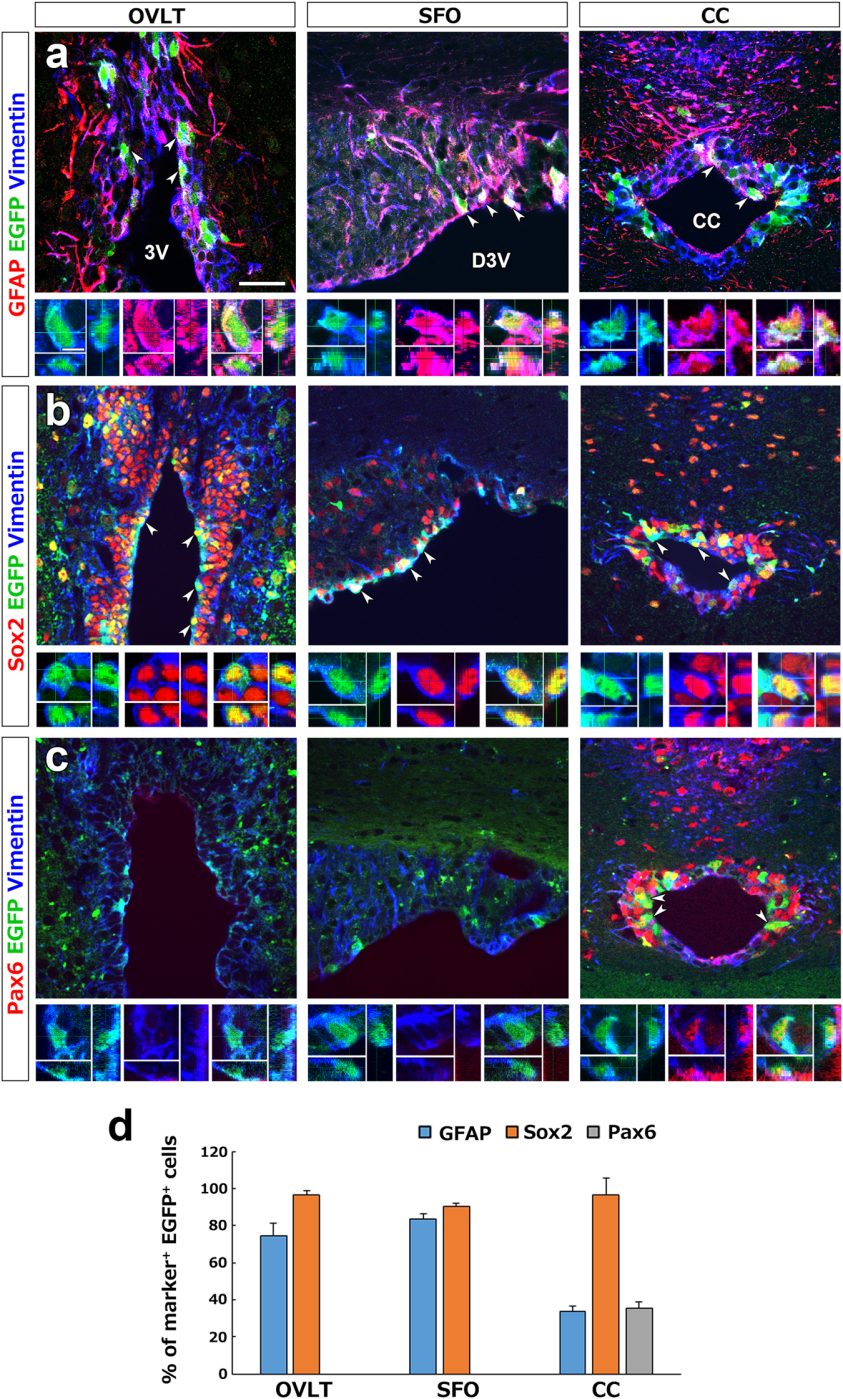
Expression profile of NSC marker proteins in EGFP^+^ tanycyte-like ependymal cells of the CVOs and the CC using transgenic adult mouse. The transgenic mice fixed at 3 days after a single intraperitoneal administration of 180 mg/kg tamoxifen. Triple labeling immunohistochemistry showed that EGFP^+^ and vimentin^+^ tanycyte-like ependymal cells (arrowheads) expressed GFAP (**a**) and Sox2 (**b**) in the OVLT, SFO and the wall of the CC, whereas the expression of Pax6 was detected only in the CC (**c**). 3D analysis of confocal images revealed the colocalization of each marker protein (bottom columns). Scale bars = 50 (top column in a), 5 (3D image in a) μm. The quantitative analysis of NSC marker protein expression showed in the OVLT and SFO that most EGFP^+^ tanycyte-like ependymal cells expressed GFAP and Sox2 but not Pax6 (**d**). In the CC, however, most of EGFP^+^ tanycyte-like ependymal cells expressed GFAP and approximately one-third of them expressed GFAP and Pax6. Data shown as mean ± s.e.m. from 4 animals. 3V, third ventricle; D3V, dorsal third ventricle.

To characterize populations of EGFP^+^ tanycyte-like ependymal cells in the OVLT, SFO and CC, we performed immunohistochemistry using NSC markers and *Nestin-CreERT2/CAG-CAT*^*loxP/loxP*^*-EGFP* transgenic mice. EGFP^+^ tanycyte-like ependymal cells often expressed glial fibrillary acidic protein (GFAP) in the OVLT and SFO, but they sometimes expressed GFAP in the CC (Fig. 4a). Three dimensional (3D) images showed the colocalization of EGFP with GFAP and vimentin in the tanycyte-like cells. Moreover, EGFP^+^ tanycyte-like ependymal cells frequently expressed Sox2 (Fig. 4b). In contrast, a subpopulation of EGFP^+^ tanycyte-like ependymal cells expressed Pax6 in the CC, but those in the OVLT and SFO never expressed Pax6 (Fig. 4c).

The quantitative evaluation of fluorescent images revealed that the GFAP^+^ percentage in EGFP^+^ tanycyte-like ependymal cell was high in the OVLT (74.23 ± 6.88%) and SFO (83.62 ± 2.97), that of the CC was low (33.65 ± 2.93) (Fig. 4d). The percentage of sex determining region Y (Sox2) expression in EGFP^+^ tanycyte-like ependymal cell was high in three examined regions (OVLT, 96.64 ± 2.00; SFO, 90.43 ± 1.90; CC, 96.64 ± 8.81). Although approximately one third (35.67 ± 3.44) of EGFP^+^ tanycyte-like ependymal cells expressed Pax6 in the CC, those of the OVLT and SFO scarcely expressed Pax6.

**Figure 5:**
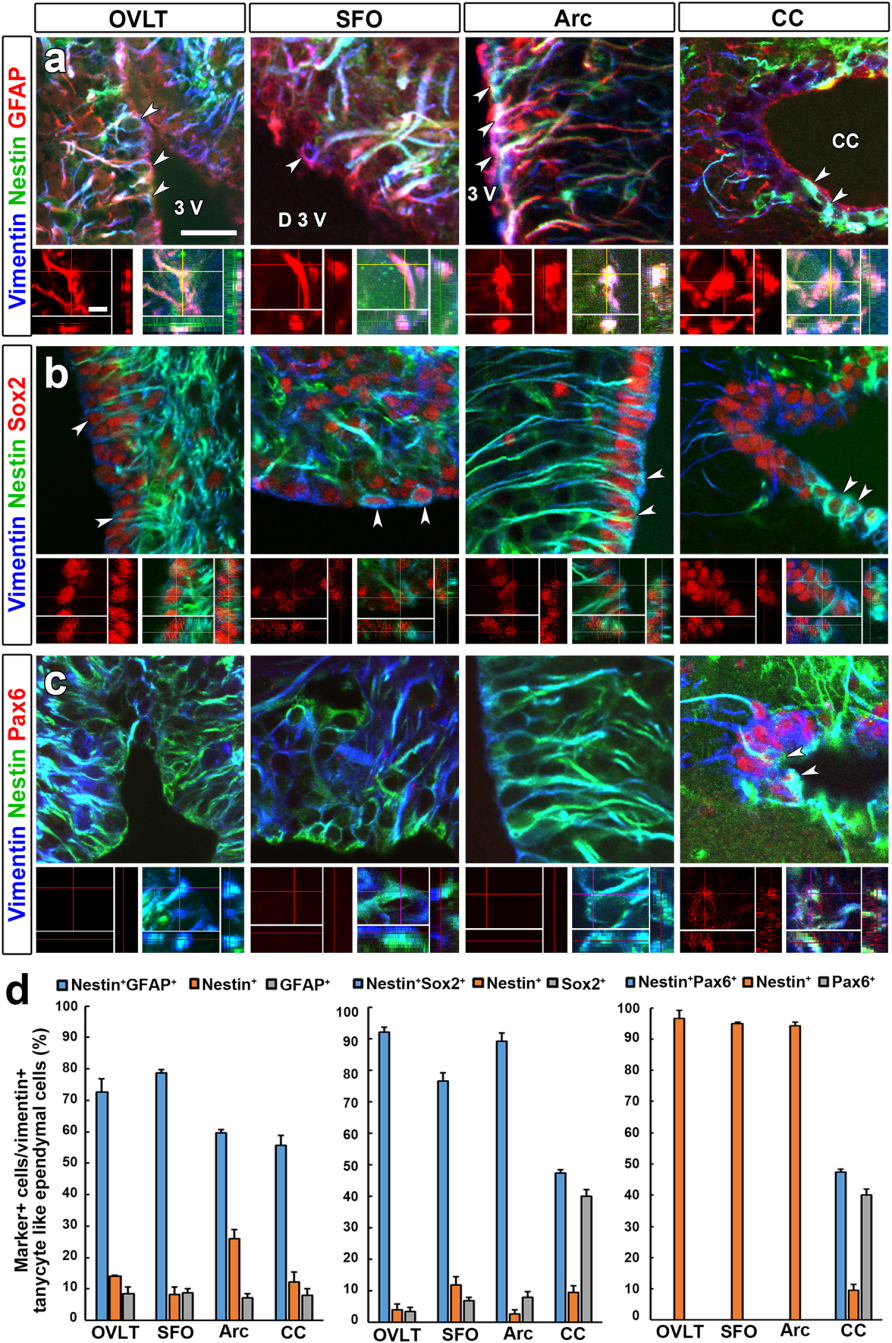
Expression profile of NSC marker proteins of tanycyte-like ependymal cells in the CVOs and the CC of wild type adult mouse. The coronal sections of C57BL/6J wild type mouse were immunostained for NSC marker proteins such as nestin, GFAP, Sox2 and Pax6 and ependymal cell marker vimentin. Majority of nestin^+^ tanycyte/tanycyte-like ependymal cells (arrowheads) expressed GFAP (**a**) and Sox2 (**b**) in the OVLT, SFO, Arc and CC. Pax6 was expressed only in the CC (**c**). Scale bars (top columns) 50 μm, (bottom columns) 5 μm. The quantitative analysis of marker^+^ cells in vimentin^+^ tanycytes/tanycyte/tanycyte-like ependymal cells in the OVLT, SFO, Arc and CC (**d**). Data shown as mean ± s.e.m. from 4 animals. 3V, third ventricle; D3V, dorsal third ventricle.

Characterization for expression of NSC marker proteins was performed in vimentin^+^ tanycyte-like ependymal cells of wild type mice. Tanycytes/tanycyte-like ependymal cells often expressed nestin and GFAP in the OVLT, SFO, Arc and CC (Fig. 5a). 3D fluorescent images revealed the colocalization of nestin and GFAP in tanycyte-like ependymal cells. Similarly, tanycytes/tanycyte-like ependymal cells frequently expressed Sox2 (Fig. 5b). Pax6 expression was observed in tanycyte-like ependymal cells only in the CC (Fig. 5c). Nestin^+^ tanycyte-like ependymal cells were seen to extend long cellular processes into brain parenchyma from their cell bodies.

The quantitative evaluation of fluorescent images showed that vimentin^+^ tanycytes/tanycyte-like ependymal cells expressed both nestin and GFAP in the OVLT (72.56 ± 4.29%) and SFO (78.79 ± 1.02) (Fig. 5d). Similarly, most of them expressed both nestin and Sox2 in the OVLT (92.24 ± 1.45) and SFO (76.73 ± 2.66) and Arc (89.43 ± 2.55). On the other hand, approximately half of vimentin^+^ tanycytes/tanycyte-like ependymal cells expressed both nestin and GFAP in the Arc (59.73 ± 1.00) and CC (55.61 ± 3.19) and both nestin and Sox2 in the CC (47.43 ± 1.04). Moreover, about half of vimentin^+^ tanycyte-like ependymal cells in the CC expressed both nestin and Pax6 (47.42 ± 1.04). These results indicate that tanycyte-like ependymal cells in the OVLT, SFO and CC morphologically resemble typical tanycytes seen in the Arc and moreover, they express typical and common NSC marker proteins.

### Proliferation of tanycyte-like ependymal cells in response to FGF-2 and EGF

**Figure 6:**
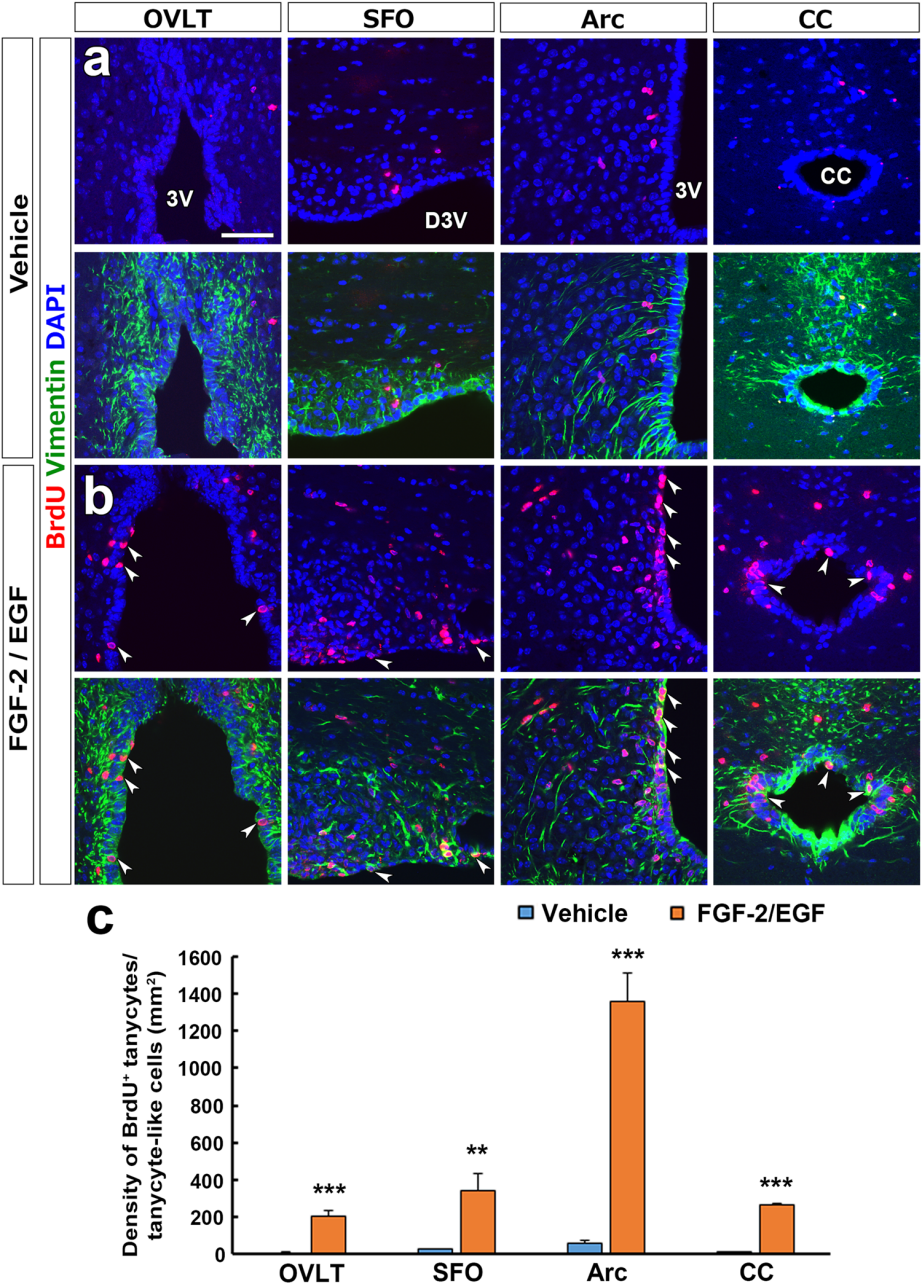
The effect of FGF-2 and EGF i.c.v. infusion on proliferation of tanycytes/tanycyte-like ependymal cells. Animals continuously received the i.c.v. infusion of FGF-2 and EGF (400 ng/day) into the right lateral ventricle using an infusion pump and BrdU via their drinking water (1 mg/ml) for 7 days and fixed for the immunohistochemistry. The chronic i.c.v. infusion of FGF-2 and EGF largely increased the number of vimentin^+^ tanycyte/tanycyte-like ependymal cells (arrowheads) in the OVLT, SFO, Arc and CC (**b**) compared with the vehicle control (**a**). The quantitative analysis revealed a significant increase in the density of BrdU^+^ tanycytes/tanycyte-like ependymal cells. Data shown as mean ± s.e.m. from 4 animals. *P < 0.05, **P < 0.01, ***P < 0.001 versus the vehicle by unpaired Student’s t test. 3V, third ventricle; D3V, dorsal third ventricle; LV, lateral ventricle.

In the SVZ, SGZ and Arc, NSCs are known to increase their proliferation and survival rate in response to FGF-2 and EGF both *in vivo* and *in vitro*^12,32,33^. The intracerebroventricular (i.c.v.) infusion of FGF-2 and EGF increased the number of BrdU^+^ vimentin^+^ tanycyte-like ependymal cells in the OVLT, SFO, Arc and CC (Fig. 6b) compared with those of the vehicle (Fig. 6a). The quantitative analysis showed that the density of BrdU^+^ vimentin^+^ tanycyte-like ependymal cells was significantly higher in the OVLT (number/mm^2^: 206.00 ± 32.77, P < 0.001), SFO (340.67 ± 93.35, P < 0.01), Arc (1359.04 ± 147.31, P < 0.001) and CC (264.63 ± 12.89, P < 0.001) of FGF-2- and EGF-treated animals than those of the vehicle (OVLT, 11.31 ± 0.99; SFO, 29.26 ± 3.89; Arc, 63.33 ± 12.50; CC, 12.72 ± 3.08) (Fig. 6c). These results indicate that tanycyte-like ependymal cells in the OVLT, SFO and CC are able to proliferate in response to FGF-2 and EGF.

### Increased proliferation of tanycyte-like ependymal cells after the collagenase-induced medullary hemorrhage

**Figure 7:**
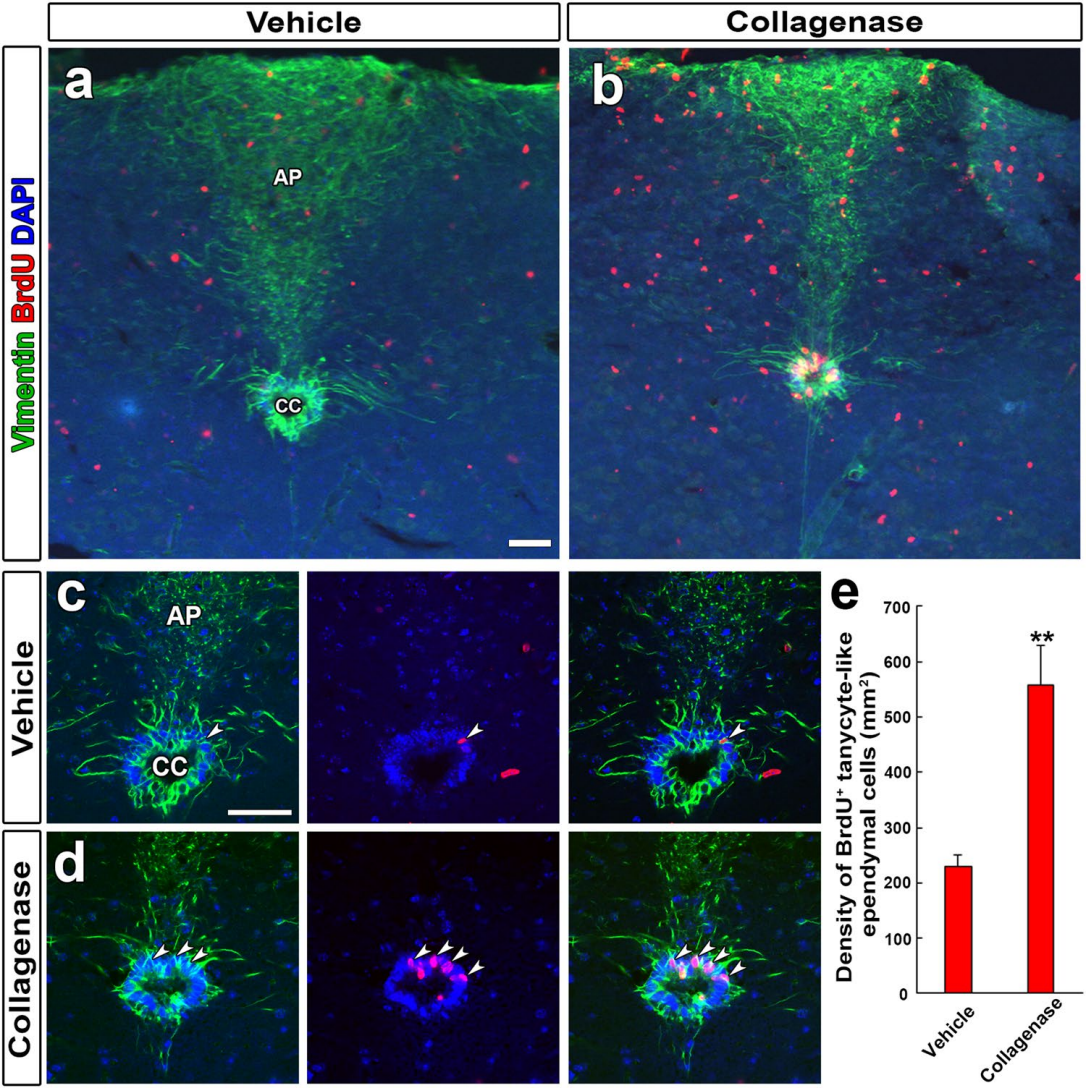
Increased proliferation in tanycyte-like ependymal cells of the CC after a collagenase-induced medullary hemorrhage. Mice received an injection of 1 µl collagenase (0.2 U/ml) into the medullary region at 1.0 mm lateral to the midline, 7.64 mm caudal to the bregma, 3.0 mm below from the brain surface and fixed at 7 days after the injection of collagenase. Mice received BrdU via their drinking water (1 mg/ml) for the same period. Many BrdU^+^ cell were increased in the medulla oblongata after the collagenase-induced hemorrhage (**b**) compared with the vehicle control (**a**). BrdU^+^ nuclei were frequently observed in vimentin^+^ tanycyte-like ependymal cells after the collagenase-induced hemorrhage (**c,d**). The quantitative analysis of BrdU^+^ and vimentin^+^ tanycyte-like ependymal cells density (**e**). Data shown as mean ± s.e.m. from 4 animals. *P < 0.05, **P < 0.01, ***P < 0.001 versus the vehicle by unpaired Student’s t test.

Activation of endogenous NSCs has been shown in the SVZ after ischemic stroke^34^ and a collagenase-induced small cerebral hemorrhage^35^. Therefore, we investigated whether proliferation of tanycyte-like ependymal cells are increased after the collagenase-induced medullary hemorrhage. Low magnification view showed that vigorous cell proliferation occurred in the medulla oblongata after the collagenase-induced medullary hemorrhage (Fig. 7a,b). High-magnification view showed the number of BrdU-labeled vimentin^+^ tanycyte-like ependymal cells was significantly increased in the CC compared with vehicle (Fig. 7c,d). The quantitative evaluation of fluorescent images showed that the density of BrdU^+^ tanycyte-like ependymal cells (number of cells/mm^2^: 705.70 ± 72.28, P < 0.01) were significantly higher in hemorrhage mice than those of the vehicle (311.32 ± 21.31) (Fig. 7e). These results demonstrate that collagenase-induced medullary hemorrhage enhances the proliferation of tanycyte-like ependymal cells in the CC of the medulla oblongata.

### Differentiation of tanycyte-like ependymal cells in the CC into astrocytes and oligodendrocytes

NSC-like characteristics such as self-renewal and multipotent capability were examined in tanycyte-like ependymal cells in the CC by employing the neurosphere assay.

**Figure 8:**
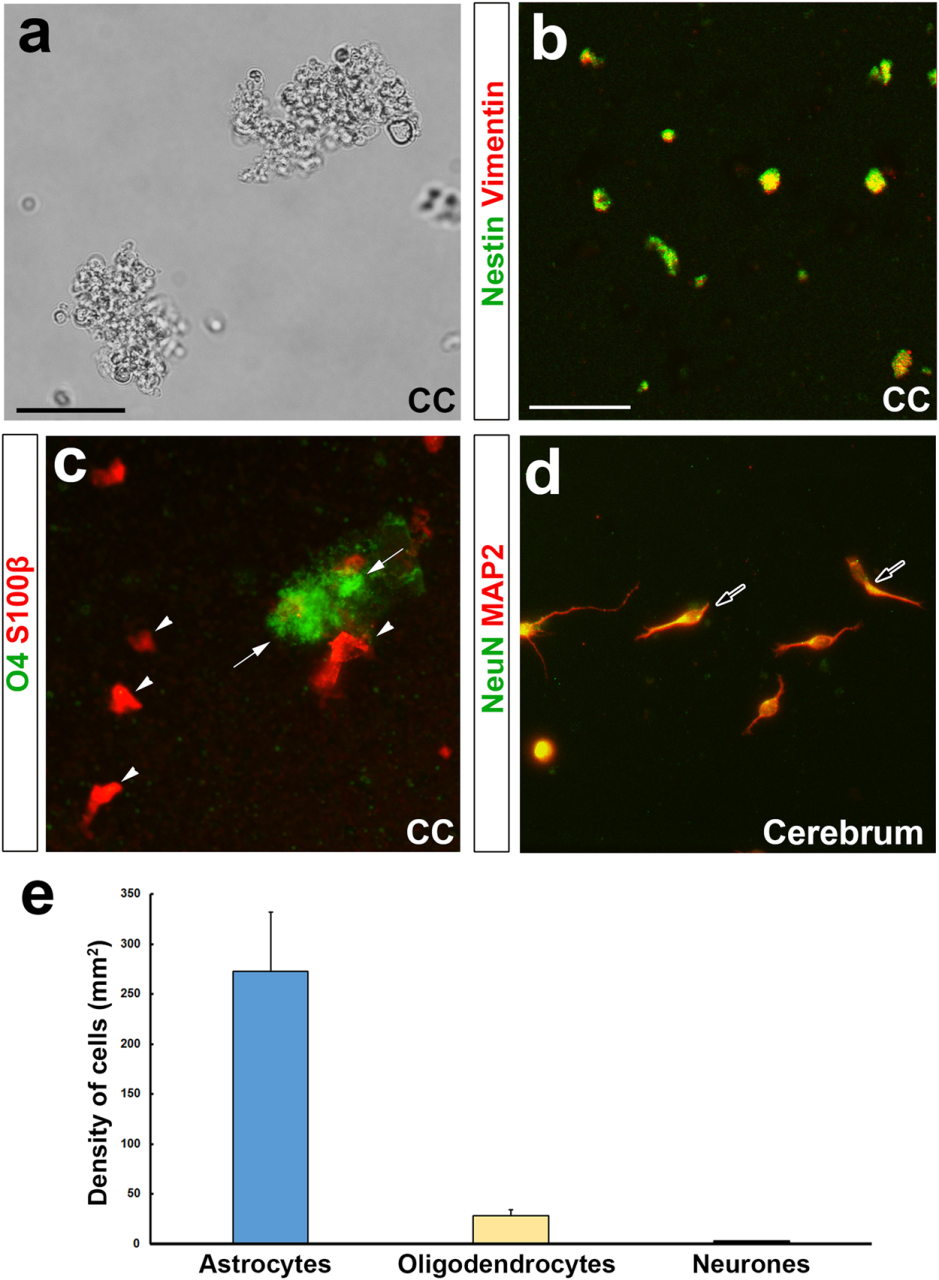
Representative images of self-renewal and multipotent capability in neurosphere culture of the CC of the medulla oblongata. Phase contrast photomicrograph shows second passage neurospheres from the CC of the medulla oblongata in 5-week old mice (**a**). Double-labeling immunocytochemistry revealed that neurosphere expressed nestin and vimentin (**b**). When dissociated neurosphere cells were seeded on Matrigel-coated glass plates with and maintained in the differentiation medium for 10 days, they differentiated into astrocytes (open arrowheads) and oligodendrocytes (open arrows) (**c**). Double-labeling immunocytochemistry of MAP2 and NeuN detected neurons (solid arrows) in the mix culture of neurons and astrocytes from the newborn mouse cerebrum (**d**). The quantitative analysis showed that neurosphere cells mainly differentiated into astrocytes and oligodendrocytes (**e**). Scale bars = 50 (**b**), 200 (**a**) μm.

In the OVLT and SFO, those area is small and astrocyte-like and tanycyte-like NSCs are localized close proximity to each other, but ependymal layer along the CC runs throughout the entire length of the medulla oblongata and is easily separated from astrocyte-like NSCs. Therefore, we performed neurosphere assay only in ependymal layer along the CC of the medulla oblongata. Neurospheres could be formed from ependymal cell layer along the CC of 5-week old mice (Fig. 8a) and dissociated neurosphere cells expressed NSC marker nestin and vimentin (Fig. 8b). After second passage, neurospheres were mechanically dissociated and plated into Matrigel- and polyethyleneimine-coated glass coverslip and cell differentiation was induced by retracting EGF and proliferation supplement from the medium. Dissociated cells differentiated into S100β-expressing astrocytes and O4-expressing oligodendrocytes (Fig. 8c). Although MAP2^+^ and NeuN^+^ neurons were often observed in a mixed culture of neurons and astrocytes from newborn mouse cerebral cortex (Fig. 8d), they were rarely seen in dissociated neurosphere cells (data not shown). The quantitative analysis showed that neurosphere cells differentiated mainly into astrocytes and minority of them gave rise to oligodendrocytes (Fig. 8e). These data show that tanycyte-like ependymal cells in the CC have the ability for self-renewal and multipotent differentiation of neural lineage cells.

## Discussion

NSCs, self-renewing and multipotent cells that generate neurons and glial cells, have been well documented to exist in the SVZ and SGZ of adult mammalian brains over the last few decades^1–3^. In addition to these well-established brain regions, it was recently proposed that tanycytes in the ME and Arc^12,13^ and astrocyte-like cells in the OVLT, SFO and AP possessed NSC niche in rodents^20–22,36^ and human^37^. In our previous study, moreover, it was presumed that tanycyte-like ependymal cells in the OVLT, SFO and CC were NSCs^22^, but it was not conclusively determined whether or not they are NSCs. In the present study, we showed prominent expression for NSC marker proteins and FGF-2 and EGF-dependent proliferation in tanycyte-like ependymal cells in the OVLT, SFO and CC of adult mouse brain. Neurosphere assay revealed that tanycyte-like ependymal cells in the CC had self-renewing and multipotent capability. Moreover, we found that collagenase-induced medullary hemorrhage increased proliferation of tanycyte-like ependymal cells in the CC. These results demonstrate that tanycyte-like ependymal cells in the CVOs and CC are NSCs and suggests that the CC probably function as stem cell source for supplying new cells against medullary damages in adult mouse.

### Tanycyte-like ependymal cells express NSC marker proteins

Tanycytes, highly specialized ependymal cells that have long cellular processes extending into parenchyma, have been described mainly in the ME/Arc of the hypothalamus^14,38^. Tanycytes are able to transport molecules between the blood and cerebrospinal fluid without the BBB leaky^14^. Tanycyte-like ependymal cells in the CVOs possess long cellular processes that extend into the parenchyma to reach the network of fenestrated capillary unlike cuboidal ependymal cells^22,24–26^. Tanycyte-like ependymal cells in the CVOs express well-organized tight junction proteins around their cell bodies and play functional diffusion barriers^26^. Thus, tanycyte-like ependymal cells in the CVOs share similar morphological and functional features with tanycytes that have been reported in the ME/Arc, but whether or not ependymal cells in the CVOs and CC possess NSC niche remains inconclusive.

Nestin, Sox2 and GFAP are common markers for NSCs and nestin is especially a useful marker, because it is expressed predominantly in NSCs of the brain and its expression is absent from nearly all mature cells in the brain^28,29^. In the present study, we showed that most tanycyte-like ependymal cells expressed nestin, GFAP and Sox2 in the OVLT, SFO and CC. The expression of nestin was also confirmed in these cells using the *Nestin-CreERT2/CAG-CAT*^*loxP/loxP*^*-EGFP* transgenic mice. These results are well coincided with protein expression pattern of these NSC marker proteins in the type-B cells of the SVZ and the type-1 cells of the SGZ^39^. On the other hand, Pax6 expression was observed in a subpopulation of tanycyte-like ependymal cells in the CC of the medulla oblongata. About 34% of Pax6^+^ cells in the SGZ expresses nestin and more than 90% of BrdU-labeled cells expresses Pax6^40^. In heterozygotes of Pax6-deficient mice, the granular cell layer becomes much thinner and the processes of GFAP^+^ radial glial cells were thinner^40^. In the present experiment, efficiency of tamoxifen-induced recombination in nestin-expressing cells at ventricular surface was higher compared with those at hippocampal parenchyma. This speculated that tamoxifen in blood more easily reach nestin-expressing cells at ventricular surface compared with those at hippocampal parenchyma. Taken together, the present study indicates that tanycyte-like ependymal cells in the OVLT, SFO and CC express typical NSCs marker proteins.

### Growth factor-dependent proliferation of tanycyte-like ependymal cells *in vivo*

The present our experiment showed that chronic i.c.v. infusion of FGF-2 and EGF promoted proliferation of tanycyte-like ependymal cells in the OVLT, SFO and CC as well as the Arc. In the present study, moreover, neurosphere assay revealed that tanycyte-like ependymal cells in the CC were able to proliferate in the presence of FGF-2 and EGF. The FGF-2- and EGF-dependent neurosphere formation has proven invaluable in demonstrating the potential to give rise to NSCs in the developing and adult central nervous system of mammals^41^. It is shown that *in vivo* infusion of FGF-2 and EGF promotes proliferation of NSCs in the SVZ^34,42^, SGZ^34^ and CC of the spinal cord in adult rodents^33^. It has been reported in the Arc of adult mouse that α-tanycyte can form neurosheres in response to FGF-2 and EGF *in vitro* and proliferation of α-tanycyte is promoted by chronic i.c.v. infusion of FGF-2 *in vivo*^12^. Taken together with NSC marker protein expression and growth factor-dependent proliferation experiments, our present results demonstrate that tanycyte-like ependymal cells in the OVLT, SFO and CC of adult mouse are NSCs.

### Tanycytes and tanycyte-like ependymal cells are NSC niche in adult brain

The present *in vitro* and *vivo* studies demonstrated that tanycyte-like ependymal cells in the OVLT, SFO and CC were NSCs. During cortical development, radial glial cells or NSCs in the ventricle zone produce new neurons and glial cells^43^. In adult central nervous system, NSCs have been considered to exist only in discrete regions of the brain, or the SGZ and SVZ where they reside in stem cell niches. On the other hand, recent evidence shows that tanycytes in the ME and Arc are neurogenic niche^12,13^. It has been shown that NSCs are present in the ependymal cell layer of the CC in the spinal cord^30,31,44^. Tanycyte-like ependymal cells in the spinal cord remarkably proliferate and produce at least astrocytes and oligodendrocytes upon spinal injury^30,31,33^. The ventricular niche for the existence of NSCs is proposed to be beneficial so that NSCs are able to receive the mechanical and chemical information from the cerebrospinal fluid to control neurogenesis and gliogenesis^45–47^. Thus, it is concluded that tanycytes and tanycyte-like ependymal cells have common feature for NSC niche throughout the adult mouse brain.

### Augmented proliferation of tanycyte-like ependymal cells or NSCs upon hemorrhage

The injury of the central nervous system is one of the major stimuli that activates quiescent NSCs.

In this study, we found tanycyte-like NSC of the CC of the medulla oblongata responds to increase their proliferation after collagenase-induced medullary hemorrhage. Furthermore, the present neurosphere assay showed that tanycyte-like ependymal cells gave rise to astrocytes and oligodendrocytes. These results are in agreement with the previous studies that proliferation of NSCs in the SVZ were promoted by collagenase-induced cerebral hemorrhage^35^. It has also been shown that NSCs in the SVZ is activated after ischemic stroke and supply cells to the striatum and cortex^34,48^. In our previous study, however, proliferation rate of astrocyte- and tanycyte-like NSCs was not increased and oligodendrocyte progenitor cells (OPCs) predominantly functioned on remyelination in the medulla oblongata in the experiments with EAE, an animal model of multiple sclerosis^36^. This is probably because supply of new oligodendrocyte at damaged region is mainly derived from resident OPCs. In our previous study, astrocyte-like NSCs in the AP also self-renewed and supplied astrocyte, oligodendrocyte and sparse numbers of neurons into adjacent medullary regions under normal condition^22^. The CC runs throughout the entire length of the medulla oblongata and spinal cord and connected with the forth ventricle^23^. Taken together with the previous and present our results, it is possible that tanycyte-like NSCs in the CC are able to increase their proliferation and may fulfil widespread supply of neural lineage cells to the overall medullary regions in response to severe medullary damage.

Conclusion: In the present study we have demonstrated that tanycyte-like ependymal cells in the OVLT, SFO and CC are NSCs. The present study also suggests that NSCs in the CC act as sources for supplying neuronal cell lineage cells to damaged brain regions. On the other hand, region-specific functions in the CVOs and CC, which are presumed to be different from those of the SVZ, SGZ and mediobasal hypothalamus and hence further study is necessary to more understand the roles of their NSCs under normal conditions.

## Materials and Methods

### Animals

Adult male mice (C57BL/6J) at 10~14-week old were used for the experiments. In some experiments, we used *Nestin-CreERT2/CAG-CAT*^loxP/loxP^*-EGFP* mice. *Nestin-CreERT2* mice^49^ were crossed with the *CAGCAT*^loxP/loxP^*-EGFP* mice^50^ to obtain *Nestin-CreERT2/CAG-CAT*^loxP/loxP^*-EGFP* animals. *Nestin-CreERT2/CAG-CAT*^loxP/loxP^*-EGFP* mice were received an intraperitoneal administration of tamoxifen (Toronto Research Chemicals, Ontario, Canada) at 180 mg/kg and fixed at 1, 2, 3 and 7 days after the tamoxifen treatment. The animals were housed in a colony room with a 12-h light/12-h dark cycle and given ad libitum access to commercial chow and tap water. Animal care and experiments were performed in accordance with the guidelines of the NIH and the Guideline for Proper Conduct of Animal Experiments by the Science Council of Japan. The experimental protocol was approved by the Animal Ethics Experimental Committee of the Kyoto Institute of Technology and Kobe University.

### Cell culture

For neurosphere culture, male mice at 5-week old were anesthesia with isoflurane and the medulla oblongata was removed after guillotine. The medulla oblongata was immersed to ice-cold saline and the CC was carefully dissected out under dissection microscope. The piece containing the CC was minced, incubated in TrypLE Express (Gibco, Thermo Fischer Scientific, Waltham, MA) for 30 min at 37 °C and then dissociated mechanically by a sequence of 25-gauge and 27-gauge needles. The single cells were obtained by passing cell strainer (size 40 μm; Corning, Corning, NY) and were plated in ultra-low binding 35 mm culture dish (EZ-BindShut SP; IWAKI GLASS, Shizuoka, Japan) in the proliferation medium consisting of NeuroCult Basal Medium (Mouse & Rat; Stem Cell Technologies, Vancouver, Canada) supplemented with penicillin/streptomycin, 10% proliferation supplement (Mouse; Stem Cell Technologies), 5% B-27 plus supplement (Gibco), 1 unit/ml heparan sulfate (Wako Chemicals, Fuji Film, Tokyo, Japan), 20 ng/ml EGF (Millipore, Temecula, CA) and 10 ng/ml FGF-2 (Cell Signaling, Danvers, MA). Neurospheres were passaged when they reach a maximal diameter typically at 7–10 days after plating. The entire cell suspension from the culture was harvested collected by centrifugation at 2,000 rpm for 5 min. Cell suspension was trituated mechanically by pipetting up and down with a 1,000 µl pipette (approximately 10 times) and resuspended in the NeuroCult proliferation medium at 5 × 10^5^~1 × 10^6^ cells/ml after the centrifugation. Single cell suspensions were plated into individual wells of a 24-well plate containing glass coverslips coated with growth factor-reduced Matrigel (Corning; dilution 1:50) and 0.1% polyethylaneimine (Sigma-Aldrich-Japan, Tokyo, Japan) after second passage. Culture medium was changed to the proliferation medium without EGF and proliferation supplement f at 5 days after plating and maintained for additional 10 days. Coverslips containing differentiated cells were removed and processed for immunocytochemistry.

For primary culture of neurons and astrocytes, newborn male mice were anesthesia with isoflurane and the cerebral cortex was removed after guillotine. The piece containing the cerebrum was dissociated as described in sphere formation except the use of DMEM medium. The cells were suspended in DMEM culture medium containing penicillin/streptomycin, 5% horse serum and 5% B-27 plus supplement (Gibco), and then plated into individual wells of a 24-well plate containing glass coverslips coated with 0.1% polyethyleneimine at 5 × 10^4^/well. Primary cultured neurons were used for immunocytochemistry 3 days after the plating.

### I.c.v. infusion of growth factor

For chronic i.c.v. administration, a stainless steel cannula (25-gauge) was implanted in each mouse under anesthesia with chloral hydrate so that its tip lays in the lateral cerebral ventricle (anteroposterior 0.3 mm, lateral 1.0 mm to bregma and dorsoventral 2.5 mm below skull) by using standard stereotaxic technique (Paxinos and Franklin, 2007). Freely moving mice continuously received i.c.v. administration of FGF-2 (Cell Signaling Technology, MA, USA; 33 μg/ml, 400 ng/day) and EGF (Millipore; 33 μg/ml, 400 ng/day) or sterilized phosphate-buffered saline (PBS) for 7 days using a Model EP-1000E infusion pump (Melquest, Toyama, Japan; 0.5 μl/h). Mice was received BrdU via their drinking water (1 mg/ml) for 7 days together with i.c.v. infusion of growth factors.

### Collagenase-induced hemorrhage

Mice received an injection of 1 µl collagenase (0.2 U/ml) into the medullary region at 1.0 mm lateral to the midline, 7.64 mm caudal to the bregma, 3.0 mm below from the brain surface by using standard stereotaxic technique (Paxinos and Franklin, 2007) and fixed at 7 days after the collagenase injection. Mice received BrdU via their drinking water (1 mg/ml) for the same 7 days just after the collagenase injection.

### Antibodies and reagents

The following primary antibodies were used in the present immunohistochemical experiments: mouse monoclonal IgG against nestin (clone Rat-401; dilution 1:100; Santa Cruz Biotechnology, Santa Cruz, CA, USA); rabbit polyclonal IgG against, green fluorescent protein (GFP: A6455, Molecular Probes; dilution 1:1000), Pax6 (PD022, Medical and Biological Laboratories; dilution 1:1000); guinea pig IgG against GFAP (YN-2011; dilution 1:400)^51^; goat polyclonal antibody against Sox2 (sc-17320, SantaCruz; dilution 1:2,000); rat IgG against BrdU (Abcam, Cambridge, UK, AB6326; dilution 1:1,000); chicken polyclonal antibody against vimentin (AB5733, Chemicon; dilution 1:12,000). For nuclear staining, sections were incubated with DAPI (1 μg/ml; Dojindo, Kumamoto, Japan).

The following primary antibodies were used in the present immunocytochemical experiments: mouse monoclonal IgG against NeuN (clone A60, Chemicon, Merck-Millipore, Temecula, CA; dilution 1:300); mouse monoclonal IgM against O4 (clone #O4, R&D Systems, McKinley Place, MN; dilution 1:1,000); guinea pig IgG against GFAP (YN-2011; dilution 1:400)^51^ and MAP2 (NH-2004; dilution 1:500)^52^.

### Immunofluorescence

For immunohistochemistry, mice were perfused transcardially with PBS (pH 7.2), containing 0.1% trisodium citrate, followed by 4% PFA in 0.1 M PB (pH 7.4) after deep anesthesia with isoflurane. After perfusion fixation, brains were dissected out, postfixed in 4% PFA in 0.1 M PB (pH 7.2) at 4 °C for 24 h, cryoprotected by 30% sucrose in PBS, and frozen quickly in Tissue-Tek OCT compound (Sakura Finetechnical, Tokyo, Japan). The sections were obtained by coronal cut on a cryostat (Leica, Wetzlar, Germany) at a thickness of 30 μm. In some experiments, they were stored at −20 °C in cryoprotectant solution (25% glycerol, 30% ethylene glycol, 45% PBS) until use^53^. For obtaining sagittal sections, brain block containing the medulla oblongata and cervical spinal cord was cut using a vibratome (DTK-1000 Microslicer, DSK, Kyoto, Japan) at a thickness of 100 μm. For immunohistochemistry, a standard immunofluorescence technique was performed on free-floating sections as described in our previous report^54^. In brief, the sections were washed with PBS and treated with 25 mM glycine in PBS for 20 min to inactivate remaining aldehyde groups. The sections were pretreated with 5% normal goat serum (NGS) in PBS containing 0.3% Triton X-100 (PBST) for 24 h at 4 °C to reduce nonspecific binding of IgG, and then incubated with the primary antibody for 48–72 h at 4 °C. They were then treated with Alexa405-, Alexa488-, or Alexa594-conjugated secondary antibody (dilution 1:400; Jackson Immunoresearch, West Grove, PA, USA) in PBST for 2 h in the case of rat, rabbit, and guinea pig primary IgG. In the case of mouse primary antibody, however, the sections were pretreated with unlabeled goat Fab fragment against mouse IgG (dilution 1:400; Jackson ImmunoResearch) for 2 h to mask endogenous mouse IgG-like proteins and Alexa488-conjugated goat F(ab)2 against mouse IgG was used (dilution 1:100; Jackson ImmunoResearch) to avoid nonspecific binding of endogenous mouse Fc receptors.

For immunocytochemistry, cultured cells were briefly washed with PBS and fixed with 4% PFA in 0.1 M PB (pH 7.5) for 10 min at 4 °C. Fixed cells were rinsed with PBS, treated with 25 mM glycine in PBS for 10 min, methanol at 20 °C for 2 min, and PBST for 15 min. After blocking with 5% NGS in PBST for overnight at 4 °C, they were then incubated with the primary antibody for 2 h at 37 °C. Cells were then treated with Alexa488- or Alexa594- conjugated secondary antibody (dilution 1:400; Jackson Immunoresearch, West Grove, PA, USA) in PBST for 2 h.

The preparations for both tissue sections and cells were sealed with Vectashield (Vector Labs, Burlingame, CA, USA) and observation was performed using laser-scanning confocal microscopes (Fluoview, FV10i; Olympus, Tokyo, Japan). Images (1024 × 1024 pixels) were saved as TIF files by employing Olympus FV10-ASW 1.7 Viewer or LSM 510 Image Browser 4.2.0.121 for Windows and arranged in Adobe Photoshop CC (Adobe Systems Incorporated, San Jose, CA).

### BrdU labeling immunohistochemistry

For BrdU labeling of proliferating cells, mice received BrdU drinking (1 mg/ml) and fixed for the immunohistochemistry. The cryosections were treated with 2N HCl at 37 °C for 20 min followed by 0.1 M borate buffer (pH 8.4) for 20 min, and then incubated with 5% NGS at 4 °C for 24 h. The sections were incubated with rat IgG against BrdU (Abcam; dilution 1:1,000) at 4 °C for 48 h and then with Alexa488- or Alexa594-conjugated goat IgG (Jackson Immuno Research Laboratories; dilution 1:400) against rat IgG in PBST for 2 h.

### Quantitative analysis and statistical analysis

We analyzed at least 5 sections for the OVLT and 7 sections for other brain regions per animal. The sections containing the following brain regions were carefully collected according to the mouse brain atlas^55^; the OVLT (between bregma 0.38 and 0.62 mm), SFO (between bregma −0.46 and −0.82 mm), AP and CC (between bregma −7.48 and −7.76 mm). For the quantitative analysis, confocal images were obtained under the same pinhole size, brightness, and contrast setting. We saved images (1024 × 1024 pixels) as TIF files by employing LSM 510 Image Browser 4.2.0.121 or Olympus FV10-ASW 1.7 Viewer for Windows, and arranged using Photoshop 7.0. The nuclei of BrdU-labeled cells were carefully identified by 3D images (interval 1 μm) using a Carl Zeiss LSM510 laser microscope. The number of BrdU-labeled cells was measured using WinRoof the threshold intensity of which was set to include measurement profiles by visual inspection and kept constant. The data were expressed as the mean ± SEM. Statistical difference was assessed using an unpaired Student’s t test.

## Supplementary information

Supplementary information
