# Supplementary material for: Neural stem cell phenotype of tanycyte-like ependymal cells in the circumventricular organs and central canal of adult mouse brain
Source: Sci Rep. 2020 Feb 18;10:2826. doi: 10.1038/s41598-020-59629-5 (PMC7029029; doi:10.1038/s41598-020-59629-5)
Supplement: Supplementary file 1 — Supplementary information [file 41598_2020_59629_MOESM1_ESM.pdf]

Supplementary Figure

Neural stem cell phenotype of tanycyte-like ependymal cells in the circumventricular organs and central canal of adult mouse brain

Eriko Furube[1,2], Haruna Ishii[1], Yuri Nambu[1], Erkin Kurganov[1], Sumiharu Nagaoka[1], Mitsuhiro Morita[3], Seiji Miyata[1]

[1]Department of Applied Biology, Kyoto Institute of Technology, Matsugasaki, Sakyo-ku, Kyoto 606-8585, Japan,[2]Department of Anatomy, Faculty of Medicine, Toho University, Omori, Ota-ku, Tokyo 143-8540, Japan,[3]Department of Biology, Graduate School of Science, Kobe University, Kobe, Japan

Supplementary Figure 1

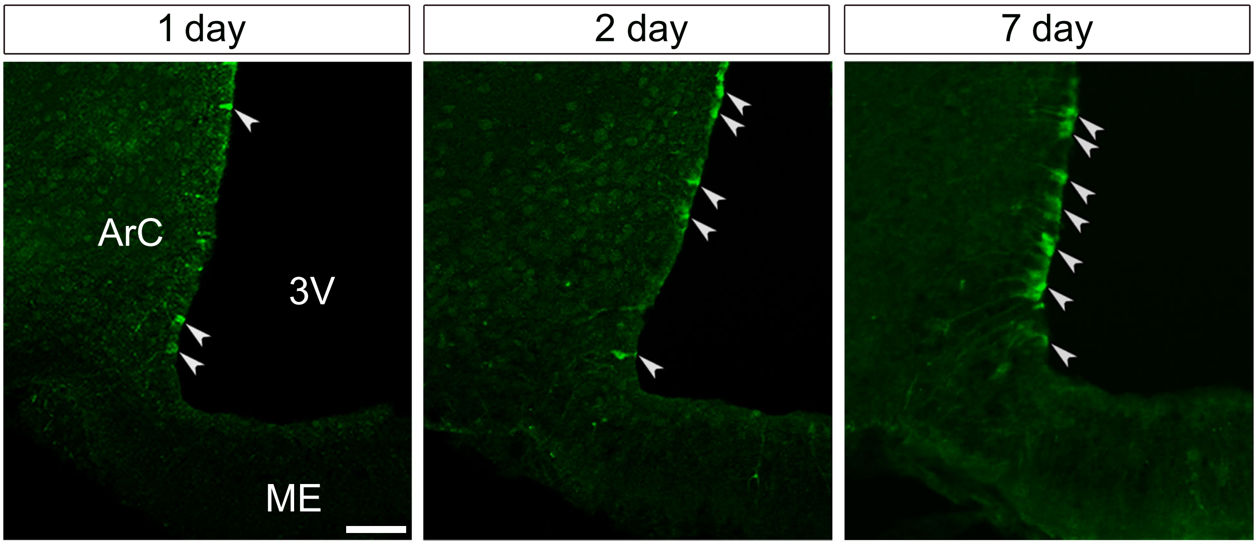

Fig. S1. EGFP+ cells in the Arc and ME of adult Nestin-CreERT2/CAG-CATloxP/loxP-EGFP mice. The transgenic mice fixed at 1, 2 and 7 days after a single intraperitoneal administration of 180 mg/kg tamoxifen. EGFP+ tanycyte-like ependymal cells (arrowheads) were seen in the Arc at 1, 2 and 7 days after the tamoxifen treatment, whereas they were scarcely observed in the ME. Scale bar = 50 μm. 3V, third ventricle.
